# Chemicals Compositions, Antioxidant and Anti-Inflammatory Activity of* Cynara scolymus* Leaves Extracts, and Analysis of Major Bioactive Polyphenols by HPLC

**DOI:** 10.1155/2017/4951937

**Published:** 2017-04-30

**Authors:** Maryem Ben Salem, Hanen Affes, Khaled Athmouni, Kamilia Ksouda, Raouia Dhouibi, Zouheir Sahnoun, Serria Hammami, Khaled Mounir Zeghal

**Affiliations:** ^1^Laboratory of Pharmacology, Faculty of Medicine of Sfax, University of Sfax, Sfax, Tunisia; ^2^Laboratory of Faculty of Sciences of Sfax, University of Sfax, Sfax, Tunisia

## Abstract

*Objective*. Artichoke (*Cynara scolymus* L.) was one of the plant remedies for primary health care. The present study was focused on the determination of chemical composition, antioxidant activities, and anti-inflammatory activity and on analyzing its major bioactive polyphenols by HPLC.* Methods*. Artichoke Leaves Extracts (ALE) were analyzed for proximate analysis and phytochemical and antioxidant activity by several methods such as DDPH, ABTS, FRAP, and beta-carotene bleaching test. The carrageenan (Carr) model induced paw oedema in order to investigate the anti-inflammatory activity. Identification and quantification of bioactive polyphenols compounds were done by HPLC method. The oxidative stress parameters were determined; CAT, SOD, GSH, MDA, and AOPP activities and the histopathological examination were also performed.* Results*. It was noted that EtOH extract of ALE contained the highest phenolic, flavonoid, and tannin contents and the strongest antioxidants activities including DDPH (94.23%), ABTS (538.75 mmol), FRAP assay (542.62 umol), and *β*-carotene bleaching (70.74%) compared to the other extracts of ALE. Administration of EtOH extract at dose 400 mg/kg/bw exhibited a maximum inhibition of inflammation induced by Carr for 3 and 5 hours compared to reference group Indomethacin (Indo).* Conclusion*. ALE displayed high potential as natural source of minerals and phytochemicals compounds with antioxidant and anti-inflammatory properties.

## 1. Introduction

The inflammation reaction is a physiologic response of the body contributed by aggression of microorganism and other soluble products. Polynuclear neutrophils (PN) play an important role in the initiation of inflammation with other molecules named inflammatory mediators released by several cells like cytokines, endotoxins, leukotrienes, prostaglandins, and reactive oxygen species (ROS) [1]. In the beginning for the inflammation reaction, the accumulation of PN in the inflammatory site and tissue and different cell injury are due to participation of proteolysis' enzymes and ROS by activating defense systems [1].

Oxidative stress is a consequence of discrepancy balance between the production of ROS and antioxidants in a system of defense of human organisms [2]. Several studies showed that many of antioxidants systems have the ability to treat some disease like a cancer by scavenging ROS resultant by oxidative stress systems [3].

Carr induced paw oedema model is used to assess the different phases of inflammation reaction. Carr model can induce acute inflammation, release of inflammatory mediators, and production of free radicals [4].

The mechanism of antioxidant enzymatic systems against the inflammatory stress includes superoxide dismutase (SOD), catalase (CAT), and nonenzymatic antioxidants as reduced glutathione (GSH). Recently, several studies showed that lack of antioxidant systems can cause many inflammatory diseases [5]. However, it showed that various roles of enzymatic and nonenzymatic antioxidants help to protect organisms from excessive generation of ROS in the inflammatory states. Some studies showed that natural herbs could suppress the production of oxidative stress by increasing the antioxidants systems [6]. Plants have rich source of phenolic compounds, carotenoids, vitamins, and terpenoids. These compounds have a potential antioxidant that can be free radical scavenger in order to reduce the development of oxidative stress in many diseases [7].

In order to enhance the Tunisian forest resources and develop new products, we are interested in the family of Asteraceae, particularly* Cynara scolymus*, which characterizes the Mediterranean region and it has been widely used in various hepatic diseases [8].

The objective of this work is designed to evaluate the phytoconstituents of ALE in vitro and the potential anti-inflammatory role of ALE in animal's models.

## 2. Materials and Methods

### 2.1. Plant Material and Extraction Method

*C. scolymus* dried leaves were obtained from the region of Bizerte in north of Tunisia; the period of collection was December to March 2014. The plant was authenticated by the Laboratory of Biology and Vegetable Ecophysiology in the Faculty of Science of Sfax. The voucher sample was created by The National Botanical Research Institute of Tunisia. Tunisia. Dried powdered plant material (200 g) was extracted by maceration method (1 L) using different increasing solvent polarities (hexane, butanol, ethyl acetate, 75% EtOH/H_2_O, and aqueous). After 48 hours, all extracts were filtered. Then the dried extracts of artichoke were kept in the dark at +4°C in order to evaluate the composition of* C. scolymus* leaves extracts.

### 2.2. Phytochemical Analysis of* Cynara scolymus* Leaves Extracts

The analytical tests for identification of different secondary metabolites in* Cynara* leaves extracts were conducted following procedures described by Sofwora and Okwu [9, 10].

### 2.3. Proximate Analysis of Dried Leaves of* C. scolymus*

The determination of nutritional composition, crude protein, lipids, fiber, and ash, was obtained using several methods described while the carbohydrate content was obtained by the difference method (calculated by subtracting the sum of crude fat, crude protein, ash content, and crude fiber) [11].

For the determination of crude protein, the concentration of dried samples was determined by micro-Kjeldahl method. The lipid content was estimated by using petroleum ether as a solvent extraction in a Soxhlet at 40–60°C [12]. Total dietary fiber was determined by extraction with petroleum ether. The defatted sample was boiled under reflux with two solvents H_2_SO_4_ and NaOH, and then they were filtered and washed with boiling water till the filtrates were no longer acidic and basic. The residue of the sample was dried in an oven at 100°C and 600°C, cooled in desiccators, and weighed [13]. The ash content was estimated by heating in a muffle furnace at 600°C.

The quantification of dry matter was performed using one gram of* C. scolymus* leaves heated in 105°C for 1 hour. Then, it was put in desiccators for 30 min. After that, the mass of each content has been noticed. These steps have led to dry leaves and their mass has been noticed again in order to calculate the percentage of humidity in these samples [11]. In addition, the analysis of sugar amounts was obtained by phenol-sulfuric acid reagent [14].

### 2.4. Quantification of Total Phenolics, Flavonoids, and Tannins Contents of* Cynara scolymus* Leaves Extracts

The quantification of total phenolics content (TPC) of ALE was determined by Fawole et al. method [15]. 200 *μ*L of ALE was mixed with 1 mL of Folin-Ciocalteu reagent diluted (×10) with distillated water and 0.8 mL of 7.5% of NaCO_3_ solution in a test tube. 30 min later, the absorbance was measured at 765 nm by using a Jenway 6405 UV-Vis spectrophotometer. TPC was expressed as milligrams of gallic acid equivalents per gram of dry weight (mg GA/g DW). Quantification of total flavonoid content (TFC) was determined spectrophotometrically [16]. 500 *μ*L of ALE was mixed with 1500 *μ*L of water and 150 *μ*L of (5%) NaNO_2_. After 5 min, 150 *μ*L of ALCl_3_ (10%, m/v) was added to mixture. After 6 min of incubation at room temperature, a volume of 500 *μ*L of NaOH (4%) was added also. Immediately, the mixture was completely agitated in order to homogenize the contents. The absorbance of solution obtained was measured at 510 nm against a blank. These analyses were expressed as mg of catechin equivalents per gram of dry weight (mg CE/g DW).

The determination of condensed tannin content (TCC) was measured using vanillin method [17]. 50 *μ*L of ALE diluted was added to mixture which was made of 3 mL of 4% methanol-vanillin solution and 1.5 mL of concentrated H_2_SO_4_. The mixture was incubated to stand for 15 min and the absorbance was measured at 500 nm against methanol as a blank. The amount of TCC was expressed as mg catechin equivalent g^−1^ dry weight (mg CE/g DW).

### 2.5. Minerals Contents Analysis

The preparation of* C. scolymus* dried leaves was incinerated in a muffle furnace at 550°C for 8 hours [18] and the ashes obtained were digested in nitric acid and dissolved in distilled water for the mineral composition of artichoke leaves [19, 20]. Minerals elements of* C. scolymus* leaves were potassium (K), magnesium (Mg), calcium (Ca), sodium (Na), iron (I), manganese (Mn), zinc (Zn), copper (Cu), and chromium (Cr). These mineral contents were determined by flame atomic absorption spectrometry (Hitachi Z-6100, Japan).

### 2.6. Analysis In Vitro of Antioxidants Properties

#### 2.6.1. Antioxidant Activity by DPPH Method

The antioxidant activity of DPPH is based on scavenging of DPPH^.^ from antioxidants in the vegetal sample, which produce a spectrophotometric loss in absorbance at 515 nm. The DPPH assay (Sigma Chemical Co., St. Louis, MO) was evaluated as described by Fawole et al. [15]. The mixture was prepared in test tubes by dilution of 50 uL of ALE in 735 mL of 100% methanol.

750 mL of 0.1 mM methanolic DPPH reagent was added to the mixture of ALE-methanol. Then, the mixture was incubated at room temperature in a chamber without any light during 30 min. After incubation, the estimation of the scavenging ability was performed by measuring at 517 nm in spectrophotometer (T70 UV-Vis).

The capacity of inhibition percentage (PI) of DPPH radicals was calculated as

(1) $$\begin{array}{c} \text{DPPH radicals (PI)}=\left[\;\frac{\left(A_{b}-A_{s}\right)}{A_{b}}\;\right]\times 100, \end{array}$$

where *A*_*b*_ refers to the absorbance of control (without plant extract) and *A*_*s*_ to the absorbance of sample (with plant extract). BHT and VC were used as standards at the same concentrations of plant extract.

#### 2.6.2. *β*-Carotene Bleaching Test

The capacity of ALE to reduce bleaching of beta-carotene was previously determined by Koleva et al. [21]. The mixture of beta-carotene and linoleic acid was prepared; 0.5 mg of *β*-carotene, 25 uL of linoleic acid, and 200 uL of Tween 40 were dissolved in 1 mL of chloroform solvent. The chloroform was evaporated in a rotator evaporator at 40°C and 100 mL of dH_2_O was added; then the mixture was stirred.

Aliquots of 2.5 mL of beta-carotene/linoleic acid emulsion obtained were transferred to test tubes containing different ALE concentrations; then the emulsion of reaction was incubated for 2 h at 50°C and the absorbance of each sample was measured at 470 nm by spectrophotometer. BHT and AA were used as the standards at the same concentrations of the samples.

#### 2.6.3. Antioxidant Activity by ABTS^•+^ Method

The ability to neutralize the ABTS^•+^ was reported by Re et al. [22] using a spectrophotometric, 96-well microplate method. The preparation of ABTS^•+^ free radical solution by incubating a mixture of ABTS (7 mM) and K_2_S_2_O_8_ (2.45 mM) dissolved in distillated water to create a stable color of radical solution following 12–16 h of incubation in the dark room at 4°C.

Therefore, the standard ABTS^•+^ solution was prepared by dilution with ethanol to a standard absorbance of 0.7 ± 0.02 at 734 nm.

50 *μ*L of plant extract (1–20 *μ*g/mL) was added to 150 *μ*L of ABTS^•+^. The plates were incubated at room temperature, in the dark room during 15 min; the absorbance was measured at 630 nm. Control wells contained 50 *μ*L of H_2_O and 150 *μ*L of ABTS^•+^. Phytochemical interference was accounted by wells containing extract (50 *μ*L) with dH_2_O (150 *μ*L), whereas 200 *μ*L of H_2_O served as blank. Trolox dissolved in pure MeOH was used as standards.

The antioxidant capacity of ALE was expressed quantitatively as mmol of Trolox Equivalents (TE) (mmol TE/g dry extract).

#### 2.6.4. Ferric Reducing Assay

The potential ability of ALE to reduce the ferric iron was evaluated previously by Fawole et al. [15].

The mixture of FRAP was freshly prepared by 50 mL of 300 mM acetate buffer, 5 mL of 10 mM TPTZ, and 5 mL of 20 mM FeCl_3_. Prior to its use, the prepared mixture must be incubated in water bath at 37°C during 15 min to stabilize the contents in the reaction mixture.

Exactly, 150 mL of diluted ALE was transferred into different test tubes and then 250 mL of FRAP solution was added in triplicate. The mixture was stirred and incubated in a dark room for 30 min before measuring the absorbance at 515 nm in UV-Vis spectrophotometer. Antioxidant capacity of ALE was expressed as micromoles of Trolox Equivalent per milliliter of sample (mMTE/mL).

### 2.7. Anti-Inflammatory Activity In Vivo

#### 2.7.1. Chemicals and Reagents

Lambda carrageenan and Indomethacin were purchased from Sigma Aldrich company (France).

#### 2.7.2. Experimental Study

30 sexually mature male rats, 10–12 weeks old weighing 150–200 g, were obtained from the Institute of Pasteur, Tunisia. Rats were fed with standard laboratory pellets and ad libitum access during the experiment study. The experimental protocol was conducted in accordance with the* Guide for the Care and Use of Laboratory Animals* issued by the University of Sfax, Tunisia, and approved by the Ethics Committee of Animal use,* protocol number 94-1939*. Before the experiment, rats were acclimatized in controlled environmental conditions at 24 ± 4°C with relative humidity (45–55%) and 12 h dark-light cycle. All the groups of animals were randomly divided into the control group and treatment groups.

#### 2.7.3. Carrageenan-Induced Paw Oedema Model

The evaluation of anti-inflammatory activity was reported by Ravi et al. [23]. 0.1 mL of Carr solution (1%) was injected into subplantar surface of the paw of each group of rats to produce acute inflammation. This method was chosen over other methods for the discovery of new therapeutic effects of ALE in inflammation diseases, as it is the most basic method requiring minimal equipment, but much practice.

The experimental study designed 4 groups of six rats. Group (I) was the control group which received isotonic saline solution Nacl (0, 9%) by subplantar injection and had no inflammation and received no treatment. Group (II) was inflamed by carrageenan injection and did not undergo any treatment (Carr). Group (III) was used as reference inflamed rats that were treated with Indomethacin (10 mg/kg/bw) (Carr + Indo) by intraperitoneal injection (i.p.) and Group (IV) was treated with EtOH extract of ALE at dose 400 mg/kg/bw (Carr+ ALE) by i.p. [24]. The doses of ALE and Indo chosen during treatments were proportional to the size of the oedema and covered the whole swelling.

In all treated groups, the oedema paw volumes, up to tibiotarsal articulation, were measured using a digital caliper at 1, 2, 3, 4, and 5 hours after Carr injection.

For each treated group, the size of oedema obtained at these various times (PT) was compared to that obtained before any treatment (P0).

Percentages of oedema inhibition were calculated as
  (2) $$\begin{array}{c} \%\;\;Inhibition\;\;\left(\;EI\;\right)=\left[\;1-\left(\;\frac{PT}{P0}\;\right)\;\right]\times 100. \end{array}$$
Percentages of inflammation inhibition were calculated as
  (3) $$\begin{array}{c} \%\;\;Inhibition\;\;\left(\;II\;\right)=\left[\;PT-\left(\;\frac{P0}{P0}\;\right)\;\right]\times 100. \end{array}$$

#### 2.7.4. Blood Sample Collection

5 hours after Carr induction, the rats were decapitated and the blood samples were collected in heparin tubes. Plasma samples were obtained after centrifugation at 3000 rpm for 15 min and they were kept in −20°C until analysis on an automatic biochemistry analyzer at the biochemical laboratory of Hedi Chaker Hospital of Sfax.

#### 2.7.5. Inflammatory Biomarkers

*(1) Determination of C-Reactive Protein (CRP).* CRP was a specific marker following the inflammatory process. It increases in proportion to its intensity [25]. The reactive protein was measured by turbid metric method using an automatic analyzer COBAS INTEGRA 400′′ C-reactive. The CRP is expressed with mg/L.

*(2) The Fibrinogen Assay of Plasma.* Plasma fibrinogen concentration was determined by Clauss clotting method [26] measured on a STA®analyzer. Principle test measures the conversion rate of fibrinogen into fibrin in diluted sample in presence of excess of thrombin and records the clotting time. The clotting time is inversely proportional to the level of fibrinogen in the plasma. The fibrinogen level is expressed with g/L plasma. 

*(3) Exploring the Antioxidant Enzymatic and Nonenzymatic Status.* Oxidative stress parameters were determined in tissues paw oedema homogenates. The supernatants obtained were removed and analyzed for the determination of MDA as described by Draper and Hadley [27]. AOPP levels were quantified by method of Kayali et al. [28]. CAT activity was measured as reported by Aebi [29] and expressed as mmoles of H_2_O_2_ consumed/(min/mg protein). SOD was assayed spectrophotometrically by colorimetric method of Beyer Jr. and Fridovich [30] and expressed as U/mg protein. GSH activity was assayed according to the method of Carlberg and Mannervik [31] and expressed as mmoles GSS/(min-mg protein) and the protein content was determined using method of Lowry et al. [32]. 

*(4) Histopathological Examination.* All the paws oedema tissues of experimental groups were collected for histological examination. First, they were fixed in 10% buffered formalin solution; second, they were embedded in paraffin wax and then cut into 5 mm thick sections and stained with hematoxylin and eosin (H&E). Finally, the slides were photographed with an Olympus U-TU1X-2 camera.

### 2.8. High Performance Liquid Chromatography (HPLC) Detection and Quantification of Polyphenolic Compounds

The phenolic fractions analysis of EtOH extract of ALE was determined using an Agilent Technologies 1100 series HPLC coupled with a UV-Vis multiwavelength detector. The separation was carried out on a 250 × 4.6 mm. C18 silica column chromatography was chosen at ambient temperature. The mobile phase consisted of C_2_H_3_N (solvent A) and water with 0.2% of H_2_SO_4_ (solvent B) and the flow rate was kept at 0.8 mL min^−1^. For the preparation of calibration curve, a standard stock solution was prepared in methanol (HPLC grade ≥ 99.9% from Sigma Chemical Company) containing Hydroxytyrosol, Tyrosol, 4-hydroxybenzoic acid, verbascoside, apigenin-7-glucoside, Oleuropein, Quercetin, Pinoresinol, cinnamic acid, and apigenin at the same concentration of 1 g/mL. For the preparation of the ethanol extract by dissolving in 1 mL of methanol making a final concentration of 25 mg/mL. Before starting HPLC analysis, all the solutions prepared were filtered with Whatman paper (Ø 0.45 *μ*m). The diluted extract was injected directly and chromatograms were monitored at 280 nm. 20 *μ*L was used for injection. Peaks were identified by the retention times compared with the standards. The analyses of phenolic profiles of ethanol extract were performed in triplicate.

### 2.9. Statistical Analysis

SPSS program was employed for comparisons between all groups. Comparison between multiple and within groups was analyzed by ANOVA followed by Tukey's tests. Values were shown as mean ± SD. The level of statistical significance was set at *p* value < 0.05. Pearson's correlation coefficient analysis was calculated using SPSS program also in order to evaluate the statistical relationship between both of polyphenolics compounds and antioxidant activities of ALE.

## 3. Results

### 3.1. Phytochemical Analysis of* Cynara scolymus* Leaves Extracts

ALE revealed the presence of flavonoid, cardiac glycosides, saponin, tannin, terpenoid, and alkaloids (Table 1).

### 3.2. Proximate Analysis of Dried Leaves of* C. scolymus*

The results showed that* Cynara* leaves contain high constituents: dry matter (97,03% DW), ash (15,81% DW), carbohydrate (80,05% DW), protein (16,64% DW), lipids (3,41% DW), total sugars (1,97% DW), and dietary fiber (71,60% DW) (Table 2).

### 3.3. Quantification of Total Phenolic, Flavonoid, and Tannins Contents of* Cynara scolymus* Leaves Extracts

The TPC, TFC, and TCC were evaluated in different solvents extracts of ALE. All results are shown in Table 3. Maximum content of TPC was obtained using EtOH extract and corresponded to 54.54 ± 1.26 mg GA/g DW followed by ethyl acetate, aqueous, and butanol extracts, while TPC of hexane extract was the lowest in comparison with EtOH extract (30.91 ± 9.36 mg GAE/g DW). The EtOH extract showed also the highest content in TFC (12 ± 0.83 CE/g DW), but this value was lower for hexane extract (8.19 ± 0.6 mg CE/g DW), respectively.

The amount of TCC which was evaluated by vanillin assay was the highest in the ethyl acetate and EtOH extract (14.51 and 14.05 mg CE/g DW), but this content was lower than aqueous extract as shown in Table 3.

### 3.4. Mineral Contents Analysis

The main composition of macroelements and microelements in* C. scolymus* leaves is presented in Table 4.

Elemental analysis in (mg/100 g of dry weight basis) indicated that leaves of artichoke contained the following order of essential minerals compounds: potassium (2886.80), sodium (1762.94), calcium (1359.34), magnesium (433.21), iron (16.17), manganese (13.05), zinc (7.37), copper (1.30), and chromium (0.12).

### 3.5. Evaluation of Antioxidant Activity

The antioxidant activity of ALE harvested in Tunisia was performed using four methods: DPPH, ABTS, FRAP, and beta-carotene test.

#### 3.5.1. Antioxidant Activity by DPPH Method

**Figure 1:**
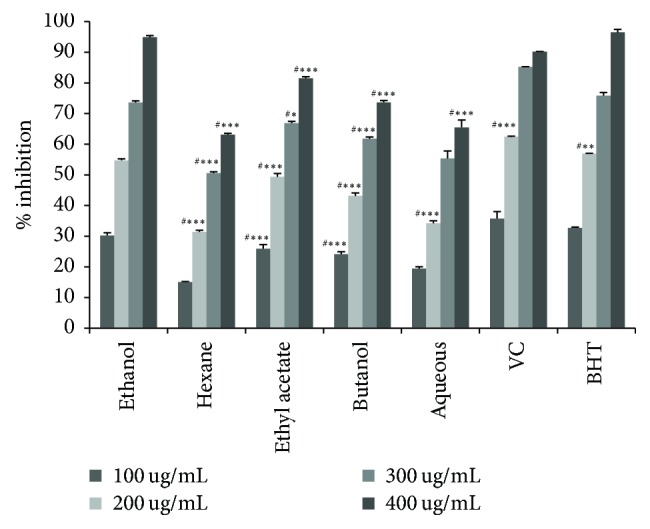
Antioxidant activity by DPPH method of* Cynara scolymus* leaves extracts at different concentrations. Values are mean ± SD (*n* = 3). ^*∗*^*p* < 0.05, ^*∗∗*^*p* < 0.01, and ^*∗∗∗*^*p* < 0.001; ^#^compared to EtOH extract. Butylated Hydroxytoluene (BHT). Vitamin C (VC).

DDPH method evaluated the capacity of compounds present in ALE to reduce DDPH radical. The result in Figure 1 showed the scavenging activity of all extracts of* C. scolymus* was concentration dependent. Among all extracts, the EtOH extract displayed the highest free radical scavenging activity at higher concentration (400 ug/mL) (94.23%) compared to the same concentration of vitamin C (9.83%) and BHT (96.23%) followed by ethyl acetate extract (81.51%); the scavenging activity of other extracts was significantly lower when compared to reference standard VC (99.83%).

#### 3.5.2. *β*-Carotene Bleaching Test

The potential effect of EtOH extract exhibited significantly the highest inhibition rate of *β*-carotene bleaching (70.74%) compared to BHT and AA (47.94%, 90.59%) (*p* < 0,001), respectively (Table 5), whereas hexane and butanol extract showed the lowest rate of *β*-carotene bleaching 37.38% and 49.39%.

#### 3.5.3. Antioxidant Activity by ABTS^•+^ Method

As evident from Table 5, ALE studied presents a potential capacity to scavenge the ABTS radical cation. Among all extracts, EtOH extract was a significantly higher value (499.43 mmol Trolox/g dry extract) than the other extracts, but the lowest level of TEAC was obtained from the hexane extract (104.3 mmol Trolox/g dry extract).

#### 3.5.4. Ferric Reducing Assay

The results in Table 5 showed that, among all extracts of ALE, a higher capacity of reducing ferric capacity was found for EtOH extract (527.79 umol Fe^2+^/mg dry extract) when compared to other extracts, respectively.

### 3.6. Anti-Inflammatory Activities

**Figure 2:**
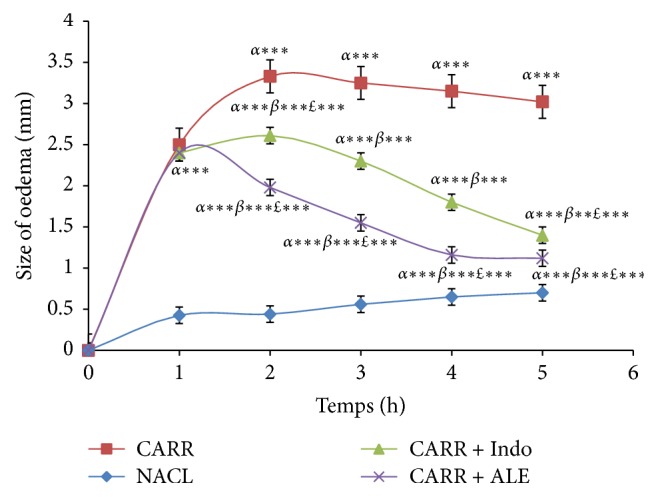
Effect of ALE and Indomethacin on paw oedema induced by carrageenan. Values represent mean ± SD (*n* = 6) in each group. ^*∗*^*p* < 0.05, ^*∗∗*^*p* < 0.01, and ^*∗∗∗*^*p* < 0.001. *α*: compared to control; *β*: compared to Carr; *£*: compared to Carr + Indo.

The appearance of Carr induced paw oedema with respective treatments groups was illustrated in Table 7 and Figure 2. The results showed that the induction of Carr in rats paw oedema started off by the vascular phase of inflammation which generated an increase in the size of oedema for all groups. This injection generated intense inflammation which peaked after 3 hours. The experimental data showed that ALE presented significant decrease in the size of paw oedema which was time dependent and more important than the reference group Indo and Carr group.

**Figure 3:**
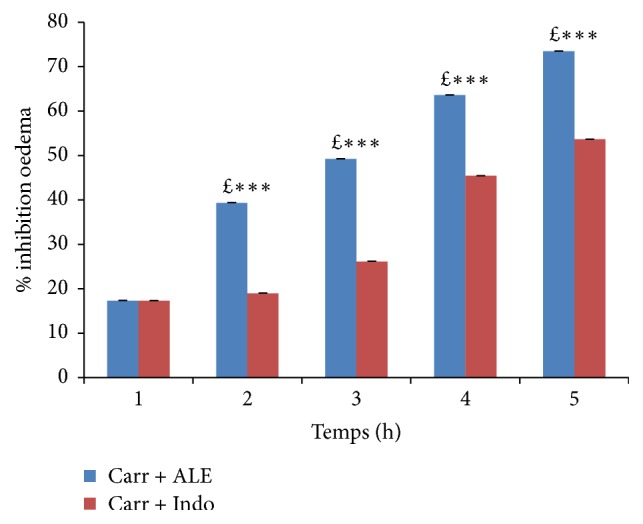
Percentage (%) of oedema inhibition data in all groups. Values represent mean ± SD (*n* = 6) in each group. ^*∗*^*p* < 0.05, ^*∗∗*^*p* < 0.01, and ^*∗∗∗*^*p* < 0.001. *£*: compared to Carr + Indo.

**Figure 4:**
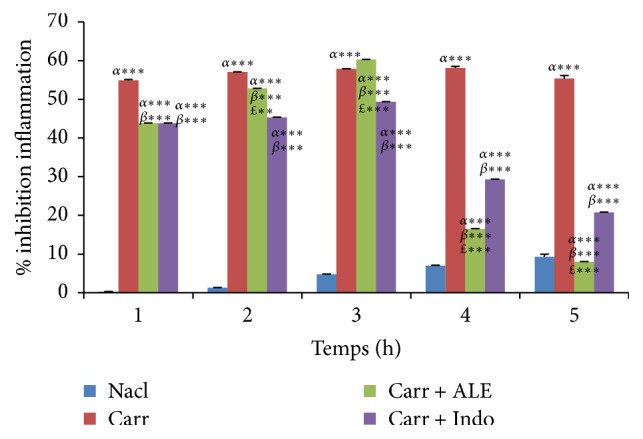
Percentage (%) of oedema inflammation data in all groups. Values represent mean ± SD (*n* = 6) in each group. ^*∗*^*p* < 0.05, ^*∗∗*^*p* < 0.01, and ^*∗∗∗*^*p* < 0.001. *α*: compared to control group, *β*: compared to Carr group, and *£*: compared to Indo group.

As seen in Figures 3 and 4, EtOH extract at dose (400 mg/kg/bw) has shown a significant percent of oedema inhibition (*p* < 0.001) in the first hour (17.3%) compared with reference group (Indo) and the significant percent of inhibition inflammation was 44,27% in comparison with the control group.

Thus, after five hours, these percentages were 735% for inhibition and 832% for inflammation. For the Indo group, oedema inhibition was significant (*p* < 0,001), 53 and 64%, and oedema inflammation was 21 and 86% in comparison with the control group.

These results showed that EtOH extract of ALE had a strong effect as Indo.

#### 3.6.1. Inflammatory Biomarkers: Fibrinogen and CRP

We assayed serum proteins of inflammatory markers in different groups of rats.

**Figure 5:**
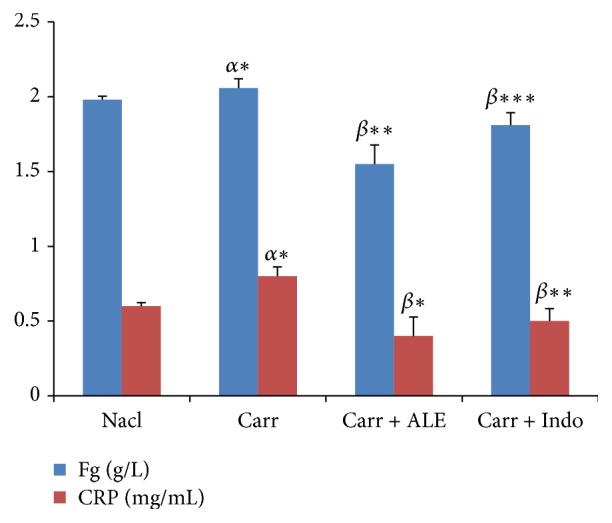
Levels of fibrinogen (Fg) and C-reactive protein (CRP). Values represent mean ± SD (*n* = 6) in each group. ^*∗*^*p* < 0.05, ^*∗∗*^*p* < 0.01, and ^*∗∗∗*^*p* < 0.001. *α*: compared to control; *β*: compared to Carr group. Fibrinogen (Fg). C-reactive protein (CRP).

*(1) Fibrinogen.* Carr group caused a significant increase in rate of fibrinogen compared to control group by 19.05% (Figure 5). The rate of fibrinogen decreased significantly (*p* < 0.01) in groups of rats treated with the EtOH extract (16.55%) and Indo (22.90%) compared with Carr group.

*(2) CRP.* Analysis showed a significant increase (*p* < 0.05) in CRP level (Figure 5) for Carr group compared to control group (16.73%). However, the injection of EtOH extract and Indo led to a significant decrease (*p* < 0,001) (60.86%, 34.23%), respectively, when compared with Carr group.

### 3.7. Oxidative Stress Parameters

MDA and AOPP levels in oedema paw were illustrated in Table 8. Results showed that Carr induced a significant increase in MDA and AOPP levels; however treatment with EtOH extract of ALE at dose of 400 mg/kg/bw showed a significant decrease (*p* < 0,001) (20.80%, 50.23%), compared with Carr group and even Indo group (10 mg/kg) (20.97%, 44.27%), respectively.

### 3.8. Exploring the Antioxidant Enzymatic and Nonenzymatic Status

CAT, SOD, and GSH levels in the paw oedema tissue of tested groups are shown in Table 8. Treatment of inflamed rats with EtOH extract restored significantly (*p* < 0.001) the SOD activity by 20.75%, the CAT activity by 56.55%, and the GSH activity by 75.66%, respectively, compared to Carr group.

### 3.9. Histopathological Examination

**Figure 6:**
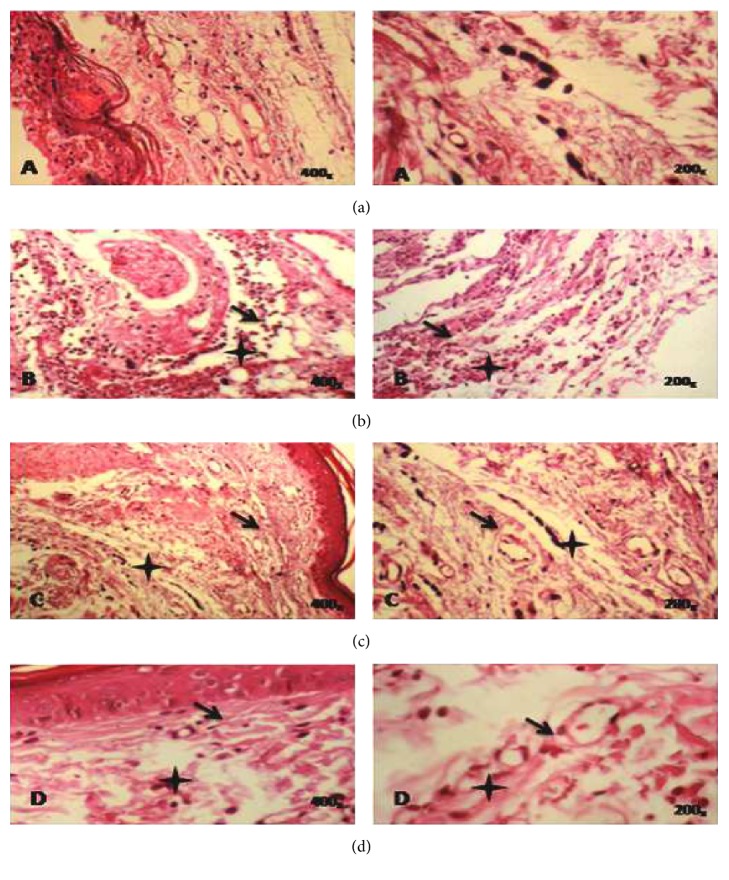
Histopathological slides tissues of paw oedema in experimental groups of rats. (a) Control group; untreated group. (b) Carr group; Carr-treated rat showed heavy infiltration of polynuclear neutrophils (PN) and a spongy-like appearance and bulla in the epidermis. (c) Carr + EtOH extract group; Carr-treated rat that received the EtOH extract (400 mg/kg/bw) reduced significantly the migration of PN and oedematasis in dermis without any spongy-like feature and bulla. (d) Carr + Indo group; Carr-treated rat that received Indomethacin (10 mg/kg/bw) showed a partial protective action. Deparaffinized hematoxylin and eosin (H&E) stained sections (200–400x). Plus sign: infiltration of PN. Arrow: odematasis in the epidermis.

A microscopic study of paw oedema tissues showed histological changes in Carr group, EtOH extract of ALE (400 mg/kg/pc) group, and Indo (10 mg/kg/bw) group (Figure 6). In the Carr group, we showed a subcutaneous oedema with infiltration of inflammatory cells, especially polynuclear neutrophils at the site of inflamed tissues, and also the presence of spongy-like appearance in the epidermis ((Figure 6(b)) as compared to control group (Figure 6(a))). The EtOH extract group produced a significant decrease in the number of cellular infiltrates and a significant reduced spongy-like appearance in the epidermis (Figure 6(c)), as did reference drug Indo (Figure 6(d)).

### 3.10. Quantification of Polyphenolic Compounds by HPLC

To the authors' knowledge, the present study identified and quantified the phenolic compounds of ALE from Tunisian origins. Accordingly, the results obtained so far by Folin-Ciocalteu needed to be further complemented to qualify phenolic constituents in EtOH extract under investigation.

**Figure 7:**
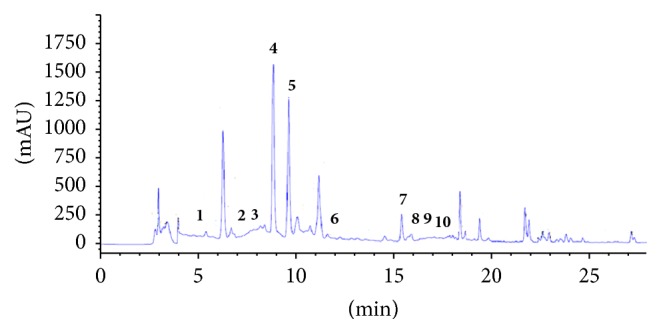
HPLC chromatogram of a standard mixture of polyphenolic compounds. Peaks:** 1**, Hydroxytyrosol;** 2**, Tyrosol;** 3**, 4-hydroxybenzoic acid;** 4**, verbascoside;** 5**, apigenin-7-glucoside;** 6**, Oleuropein;** 7**, Quercetin;** 8**, Pinoresinol;** 9**, cinnamic acid;** 10**, apigenin.

**Figure 8:**
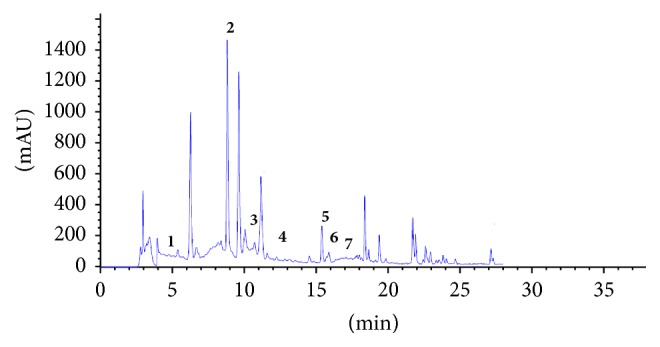
HPLC chromatogram of ethanol extract of* Cynara scolymus leaves extracts*. Peaks:** 1**, Hydroxytyrosol;** 2**, verbascoside;** 3**, apigenin-7-glucoside;** 4**, Oleuropein;** 5**, Quercetin;** 6**, Pinoresinol;** 7**, apigenin.

The findings revealed the presence of seven phenolic compounds, namely, Hydroxytyrosol, verbascoside, apigenin-7-glucoside, Oleuropein, Quercetin, Pinoresinol, and apigenin. HPLC analysis indicated that ALE have significant amount of verbascoside content 38,1 mg/100 g and Quercetin content (19, 2 mg/100 g) (Figures 7 and 8 and Table 9).

### 3.11. Correlation between the Polyphenolic Compounds and Antioxidant Capacity

In order to determine the contribution of the phenolic and flavonoid content in ALE on antioxidant capacity, the Pearson correlation coefficient (*r*) was determined in Table 6. The results showed a significant linear correlation between antioxidant activities determined by using DPPH, ABTS, FRAP, and beta-carotene methods and TPC and TFC, respectively. The strongest correlative value is obtained with DDPH^.^ and TPC (*r* = 0.87). These results indicated a good correlation between TPC and TFC with antioxidant activities of ALE.

## 4. Discussion

The importance of plants in traditional medicine remedies and the potential of phytochemical constituents were discussed nowadays with respect to their benefit in pharmacotherapy in Tunisia.* Cynara scolymus* was one of these medicinal plants which have a beneficial potential effect attributed to its source of polyphenolic compounds [3].

The selection of this plant was guided by the indications of its traditional use that, at present, there have been very little chemical and biological investigations done. Therefore the present study was to investigate phytochemical composition and their antioxidant capacity and to evaluate in vivo the anti-inflammatory effects of* Cynara scolymus* leaves extracts.

The presence of phenolic compounds is very widely distributed in medicinal plants; several studies showed that these compounds have drawn much attention to their potential antioxidant abilities which demonstrated their beneficial implications for human health.

Regarding the determination of phenolic compounds, the EtOH extract of ALE had the highest amount of these compounds in comparison with other extracts, which were an agreement with the result of Emanue et al. [33], who shows that EtOH extract exhibited the maximum amount of phenolic compounds from the leaves of* Cynara scolymus*, whereas it differs from the reports of Oliveira et al. [34], who proves that aqueous extract of ALE is the most suitable solvent for extraction of phenolic compounds.

The importance of TPC and TFC extraction yields obtained with EtOH extract can be attributed to its good solubility, low toxicity, medium polarity, and high extraction capacity [35].

In the first investigation of my study, five solvents of increasing polarities were chosen for the determination of phenolic compounds from leaves of* C. scolymus*, namely, hexane, ethyl acetate, butanol, 75% EtOH/H_2_O, and aqueous, in order to determine the variability of TPC and TFC in the aerial part of* Cynara* as a function of each extraction solvent.

This variability of phenolic compounds in ALE can be attributed to the wide range of solubility displayed by various polar compounds within the ALE solvents, the degree of polymerization of phenols and their interaction, genetic factors, geographical variations, and climatic changes [36].

Overall, these findings indicate that EtOH extract of ALE was rich in phenolic and flavonoid contents, which could be the major contributor to their antioxidative properties. Many researches revealed that flavonoids and polyphenols displayed the highest ability of scavenging activity in medicinal plants [37, 38].

The results indicate that EtOH extract of ALE, which contains the highest content of TFC and TPC, displays the highest free radical scavenging activity (94.23%) at concentration of 400 ug/mL. However, Oliveira et al. [34] show that aqueous extract of ALE has good radical scavenging (83.40%) at concentration of 200 ug/mL. Furthermore, our results suggest that some components within EtOH extract are significantly the strongest radical scavenger in comparison with other extracts. The TEAC values of ALE showed also that EtOH extract presents a potential ability to scavenge the ABTS radical cation in accordance with the report of Betancor-Fernández et al. [39].

The FRAP assay shows also that EtOH extract has the highest values (542.62 umol Fe II g/DW). The same result is confirmed by Kukić et al. [40]. Therefore, the results of positive correlation between the TPC, TFC, and the antioxidant methods in vitro suggest that phenolic compounds act as reducing agents, hydrogen donors, and singlet oxygen scavengers [3] and may exert an important in vitro antioxidant capacity of ALE.

The phytochemical screening of ALE has been found to be a rich source of polyphenolic compounds including Quercetin, apigenin-7-glucoside, and verbascoside. These compounds have shown a great potential of antioxidant activity [41].

The research into medicinal plants used as pain relievers' agents should therefore be viewed as new therapy in the inflammatory diseases [42]. The anti-inflammatory activities of EtOH extract of ALE are investigated, applying in vivo experimental Carr model compared with a nonsteroidal anti-inflammatory (Indomethacin); this drug was reported by Higgs et al. [43] who demonstrated the role of Indo to inhibit the biosynthesis of prostaglandins in Carr model rats. The experimental study of anti-inflammatory activity is performed by the Carr test. This phlogiston agent induced tissue oedema characteristic of acute inflammation which is regarded as a crucial parameter in assessing anti-inflammatory activity [44].

The Carr injection produces biphasic states; in the first phase [45], there is an increase in the mRNA synthesis of cyclooxygenase-2 (COX-2) at the first hour. This increase is accompanied by an amplification of synthesis of strong proinflammatory mediator such as prostaglandin, especially the prostaglandin type 2 involved in the inflammatory process [46], serotonin, bradykinin, and leukotrienes, which contribute to the initiation of inflammation reaction. Moreover, the participation of arachidonic metabolites is the main factor responsible for both of phases of Carr induced inflammation.

In the second phase, the increase of vascular permeability is observed by release of kinins during 2.30 h. Thereafter from 2.30 h to 5 h, inflammation is mediated by prostaglandins and is also associated with a release infiltration and migration of PN into the inflamed site [47].

Our results indicate that ALE affords protection against the Carr induced acute inflammation in dose dependent manner. EtOH extract of ALE at dose of 400 mg/kg/bw exhibits significant anti-inflammatory activity with 73% inhibition of paw oedema compared with Indo group (53%) and typically reaches a maximum at 5 h; these results obtained suggest the anti-inflammatory effect of EtOH extract of ALE by means of inhibiting the synthesis and the release of inflammatory mediators like histamine, serotonin, and prostaglandins that are involved in acute inflammation. These results revealed the inhibitory effect of EtOH extract on PN migration and it is confirmed through a histological analysis of tissues of paw oedema in experimental groups.

These findings clearly confirm that EtOH extract of ALE has an anti-inflammatory effect by reducing the influx of polymorphonuclear cells to inflammatory tissue following injection of Carr. Most inflammatory markers, such as interleukin-6 (IL-6), tumor necrosis factor-*α* (TNF-*α*), CRP, and fibrinogen, increased significantly in response to infection and in active diseases states. CRP is the specific marker of acute inflammation occurring in the body [48]; moreover the fibrinogen is the most relevant indicator of inflammation, and it does not only represent the acute-phase reactant that is increased in inflammatory states. Elevated fibrinogen levels would be associated with higher levels of CRP and would similarly correlate with inflammation reaction.

Therefore the present study showed that EtOH extract of ALE significantly decreases the level of fibrinogen and CRP by value of 22.72% and 34.78% in comparison with Carr group.

The induction of inflammation with Carr is manifested by generation of ROS and has been shown to play an important role in various forms of inflammation [49, 50]. High concentration of reactive free radicals contributes to lipid peroxidation and protein oxidation [3]. Our results showed that there is a significant increase in MDA and AOPP activities in Carr group, when compared to control group, and a highly significant decrease in activities of SOD, CAT, and GSH, also observed at the tissue level compared with the control group (*p* < 0.001). However, the injection of EtOH extract and Indo shows a significant decrease of the levels of MDA and AOPP by 34.27% and 44.27% following a significant increase in antioxidant activities as CAT, SOD, and GSH when compared to Carr group (20.16%, 73.30%, and 75%). From these results, it is implied that the protective effect of EtOH extract may be attributed to its potential indirectly as a stimulator of the activity and expression of antioxidant enzymes during inflammation.

Thus, the anti-inflammatory activity is due to the individual or synergistic effect of the components in the ALE. In fact, previous studies have found that anti-inflammatory profile of ALE could be related to polyphenolic compounds according to the phytochemical analysis. We performed HPLC analysis to justify the correlation between phytochemical compounds and anti-inflammatory activity of this plant. Among these polyphenolic compounds, verbascoside has been reported in several reports and showed a potential spectrum of many activities including antioxidant and anti-inflammatory. Particular, verbascoside presents antiedematogenic activities in animal models of Carr induced inflammation [51] and the responses exhibited the maximum activity for the fourth hour of treatment. Such activities may be derived from inhibitory action of chemical mediators of the inflammatory process such as histamine and bradykinin. A significant antioxidant effect of verbascoside has been recently reported by Aleo et al. [52] in an experimental study [53]. Other authors reported that anti-inflammatory activity of verbascoside has been evaluated by an in vitro test performed on cell cultures of primary human keratinocytes [54] in which it was able to reduce the release of proinflammatory markers such as chemokines.

Apigenin-7-glucoside was found to block the release of several varieties of enzymes involved in inflammation including especially lipoxygenases and cyclooxygenases [55, 56] leading to inhibition of proinflammatory molecules NF-kB activation and inhibits neutrophil infiltration in tissues.

From these results, we suggest that anti-inflammatory activity observed is due to a synergic action of these phenolic components contained in EtOH extract of ALE.

*C. scolymus* is one of the few herbal remedies which have been verified through experimental studies. Further the toxicity studies of ALE are required in order to confirm the safety of this medicinal plant in the treatment of inflammation diseases.

## 5. Conclusion

The present study showed clearly the advantages of* C. scolymus* leaves extracts which have safer anti-inflammatory profile with potent antioxidant activity attributed to the phenolic compounds. Furthermore, in the future studies, we are interested to characterize the action mechanisms of active phenolic compounds of ALE responsible for anti-inflammatory and antioxidants activities.

## Figures and Tables

**Table 1:** Phytochemical analysis of *Cynara scolymus *extracts leaves.

Extract	Cardiac glycosides	Triterpenoids	Saponin	Flavonoids	Tannins	Alkaloid
ALE	+	+	+	+	+	+

Sign (+) indicates being present.

**Table 2:** Proximate analysis of dried leaves of *Cynara scolymus.*

Constituents	*Cynara scolymus* leaves (percentage dry weight basis)
Dry matter	97.03 ± 0.43
Ash	15.81 ± 0.01
Carbohydrate	80.05 ± 0.69
Protein	16.64 ± 1.79
Lipids	3.41 ± 0.45
Total sugars	1.97 ± 0.10
Dietary fiber	71.60 ± 0.81

Values are expressed as mean ± SD (*n* = 3).

**Table 3:** Quantification of total phenolic, flavonoids, and tannins contents of *Cynara scolymus* leaves extracts.

Extracts	Phenolics content (mg GAE/g DW)	Flavonoids content (mg CE/g DW)	Tannins content (mg CE/g DW)
Hexane	39.91 ± 9.36	8.19 ± 0.16	14.05 ± 0.3
Ethyl acetate	53.07 ± 0.47	10.32 ± 0.12	14.51 ± 0.13
Butanol	41.66 ± 2.23	11.21 ± 0.10	13.93 ± 93
Ethanol	54.54 ± 1.26	12.00 ± 0.83	10.99
Aqueous	49.49 ± 0.39	9.49 ± 0.39	4.38 ± 0.45

Values are expressed as mean ± SD (*n* = 3).

**Table 4:** Mineral contents dried leaves of *Cynara scolymus*.

Elements	*Cynara scolymus* leaves (mg/100 g of dry weight basis)
K	2886.803 ± 12.0
Ca	1359.346 ± 5.05
Na	1762.946 ± 12.0
Mg	433.219 ± 23.4
I	16.176 ± 0.14
Mn	13.051 ± 0.11
Zn	7.371 ± 0.14
Copper	1.30 ± 0.16
Cr	0.124 ± 0.01

Values are expressed as mean ± SD (*n* = 3).

**Table 5:** Antioxidant activities of *Cynara scolymus* leaves extracts.

Sample	FRAP assay (umol Fe (II)/g DW)	*β*-Carotene bleaching assay (%)	TEAC assay (mmol Trolox/g DW)
Hexane	223.023 ± 11.16^#*∗∗∗*^	37.38 ± 5.24^#*∗∗∗*^	104.3 ± 10.73^#*∗∗∗*^
Ethyl acetate	508.29 ± 5.24^#*∗∗∗*^	61.56 ± 8.17^#*∗*^	382.60 ± 5.24^#*∗∗*^
Butanol	443.06 ± 22.98^#*∗∗∗*^	49.393 ± 2.24^#*∗∗*^	251.93 ± 28.15^#*∗∗∗*^
Ethanol	527.79 ± 16.26	70.743 ± 1.29	499.43 ± 39.72
Aqueous	315.91 ± 8.36^#*∗∗∗*^	56.11 ± 5.43^#*∗∗*^	210.74 ± 8.36^#*∗∗∗*^
BHT	—	47.94 ± 0.75^#*∗∗∗*^	—
AA	—	90.59 ± 3.25^#*∗∗∗*^	—

Values are means ± SD (*n* = 3). Butylated Hydroxytoluene (BHT) and Ascorbic Acid (AA) were used as positive control; ^#^compared with EtOH extract. ^*∗*^*p* < 0.05, ^*∗∗*^*p* < 0.01, and ^*∗∗∗*^*p* < 0.001.

**Table 6:** Pearson correlation coefficient (*r*) between the content of phenolic compounds and antioxidant capacity (DDPH^•^, ABTS^•+^, FRAP, and beta-carotene).

Correlation	*R*
TPC versus DDPH^•^	0,870^*∗∗*^
TPC versus ABTS^•+^	0,848^*∗∗*^
TPC versus FRAP	0,707^*∗∗*^
TPC versus beta-carotene	0,842^*∗∗*^
TFC versus DDPH^•^	0,728^*∗∗*^
TFC versus ABTS^•+^	0,743^*∗∗*^
TFC versus FRAP	0,849^*∗∗*^
TFC versus beta-carotene	0,712^*∗∗*^

DPPH: 2,2-diphenyl-1-picrylhydrazyl; ABTS: 2,20-azinobis(3-ethylbenzothiazoline-6-sulphonic acid) diammonium salt; FRAP: ferric reducing; TPC: total phenolic content; TFC: total flavonoid content; ^*∗∗*^*p* < 0.01 significant correlation.

**Table 7:** Effect of *Cynara scolymus* leaves extract on carrageenan-induced rat paw oedema.

Treatment	Oedema size (mean ± SD) (mm)					
	0 h	1 h	2 h	3 h	4 h	5 h
Control	0.426 ± 0,00	0.441 ± 0.01	0.56 ± 0.03	0.650 ± 0.05	0.755 ± 0.05	0.70 ± 0.01
Carr	0.426 ± 0,00	2.8 ± 0.26^*α∗∗∗*^	3.33 ± 0.28^*α∗∗∗*^	3.25 ± 0.02^*α∗∗∗*^	3.15 ± 0.01^*α∗∗∗*^	3.02 ± 0.09^*α∗∗∗*^
Carr + Indo (10 mg/kg)	0.426 ± 0,00	2.46 ± 0.26^*α∗∗∗β∗*^	2.61 ± 0.07^*α∗∗∗β∗∗∗*^	2.30 ± 0.02^*α∗∗∗β∗∗∗*^	1.8 ± 0.11^*α∗∗∗β∗∗∗*^	1.4 ± 0.06^*α∗∗∗β∗∗∗*^
Carr + ALE (400 mg/kg/bw)	0.426 ± 0,00	2.480 ± 0.05^*α∗∗∗β∗∗∗*^	1.88 ± 0.04^*α∗∗∗β∗∗∗£∗∗∗*^	1.65 ± 0.05^*α∗∗∗β∗∗∗£∗∗∗*^	1.16 ± 0.20^*α∗∗∗β∗∗∗£∗∗∗*^	1.12 ± 0.1^*α∗∗∗β∗∗∗£∗∗∗*^

Values represent means ± SD (*n* = 6) in each group. ^*∗*^*p* < 0.05, ^*∗∗*^*p* < 0.01, and ^*∗∗∗*^*p* < 0.001.

*α*: compared to control; *β*: compared to Carr; £: compared to Carr + Indo.

**Table 8:** Effects of *Cynara scolymus *leaves extract and Indomethacin on CAT, SOD, GSH, AOPP, and MDA activities in carrageenan induced paw oedema.

Groups	CAT (*μ*moles CAT/min/mg protein)	SOD (unit SOD/min/mg protein)	GSH (nmoles/mg protein)	MDA (nmol MDA/mg protein)	AOPP (nmol/mg protein)
Control	47 ± 2	13.1 ± 4.3	42.1 ± 1.2	23.4 ± 5	48.4 ± 4.3
Carr	16.2 ± 0.5^*α∗∗∗*^	15.3 ± 2.6^*α∗∗∗*^	13.1 ± 1.1^*α∗∗∗*^	31.4 ± 7.5^*α∗∗∗*^	67.9 ± 6.9^*α∗∗∗*^
Carr + Indo (10 mg/kg/bw)	64.9 ± 7.1^*β∗∗∗*^	24.0 ± 1.3^*β∗∗∗*^	70.3 ± 1.1^*β∗∗∗*^	20.9 ± 3.0^*β∗∗∗*^	44.2 ± 0.6^*β∗∗∗*^
Carr + ALE (400 mg/kg/bw)	56.5 ± 2.5^*β∗∗∗£∗∗*^	20.7 ± 4.0^*β∗∗∗£∗∗*^	75.6 ± 2.5^*β∗∗∗£∗*^	20.8 ± 6.0^*β∗∗∗*^	50.2 ± 4.1^*β∗∗∗£∗*^

Values represent means ± SD (*n* = 6) in each group. ^*∗*^*p* < 0.05, ^*∗∗*^*p* < 0.01, and ^*∗∗∗*^*p* < 0.001.

*α*: compared to control; *β*: compared to Carr; *£*: compared to Carr + Indo.

SOD: superoxide dismutase.

CAT: catalase.

GSH: glutathione peroxidase.

MDA: Malondialdehyde.

AOPP: Advanced Oxidation Protein Product.

**Table 9:** Quantification and identification of phenolic compounds contents in the ethanol extract of *Cynara scolymus* leaves extracts.

Number	Content (mg/100 g of dry extract)	Polyphenolic compound
**1**	2,8 ± 0,19	Hydroxytyrosol
**2**	38,1 ± 0,36	Verbascoside
**3**	9,9 ± 0,02	Apigenin-7-glucoside
**4**	18 ± 0,1	Oleuropein
**5**	19,2 ± 0,14	Quercetin
**6**	5 ± 0,05	Pinoresinol
**7**	4 ± 0,04	Apigenin

Values represent means ± SD (*n* = 3).
